# A Rapidly Growing Forearm Pilomatricoma in an Elderly Patient

**DOI:** 10.7759/cureus.39043

**Published:** 2023-05-15

**Authors:** Kristina Blegen, Michelle Samaniego, Cloyce Stetson, Ashley Sturgeon

**Affiliations:** 1 Department of Dermatology, Texas Tech University Health Sciences Center, Lubbock, USA; 2 Department of Dermatology, Texas Tech University Health Sciences Center El Paso, Paul L. Foster School of Medicine, El Paso, USA

**Keywords:** calcified epithelial carcinoma, pilomatrical carcinoma, calcifying epithelioma of malherbe, pilomatrixoma, pilomatricoma

## Abstract

Pilomatricoma is a benign skin tumor of epithelial hair matrix cells that typically presents as a solitary nodule on the head or upper trunk. It occurs most often in children and young adults. While considered uncommon in middle-aged and elderly patients, there are reports of elderly patients with histopathologically diagnosed pilomatricomas; however, these cases primarily occurred on the face. We present a case of an 88-year-old female with a history of non-melanoma skin cancer who presented with a new, rapidly enlarging, biopsy-proven pilomatricoma on the forearm. This case highlights a unique age of onset and location for this skin tumor, suggesting that pilomatricomas are not limited to children and young adults and should be considered in the differential diagnosis of rapidly growing skin lesions in elderly patients. Diagnosis should be confirmed with biopsy in elderly patients, as pilomatricomas may mimic malignant skin lesions.

## Introduction

Pilomatricoma, also known as calcifying epithelioma of Malherbe, is a benign skin tumor of epithelial hair matrix cells [1]. It presents clinically as a solitary, skin-colored, erythematous or bluish nodule on hair-bearing skin, usually the head or upper trunk, in children or young adults [2]. Pilomatricomas have been considered uncommon in middle-aged and elderly patients [3]. Here, we present a case of an 88-year-old female with a new, rapidly enlarging pilomatricoma on the forearm, highlighting a unique age of onset and location for this skin tumor.

This article was previously presented as a poster abstract at the Texas Dermatological Society Spring 2023 Annual Conference, April 28-29, 2023.

## Case presentation

**Figure 1:**
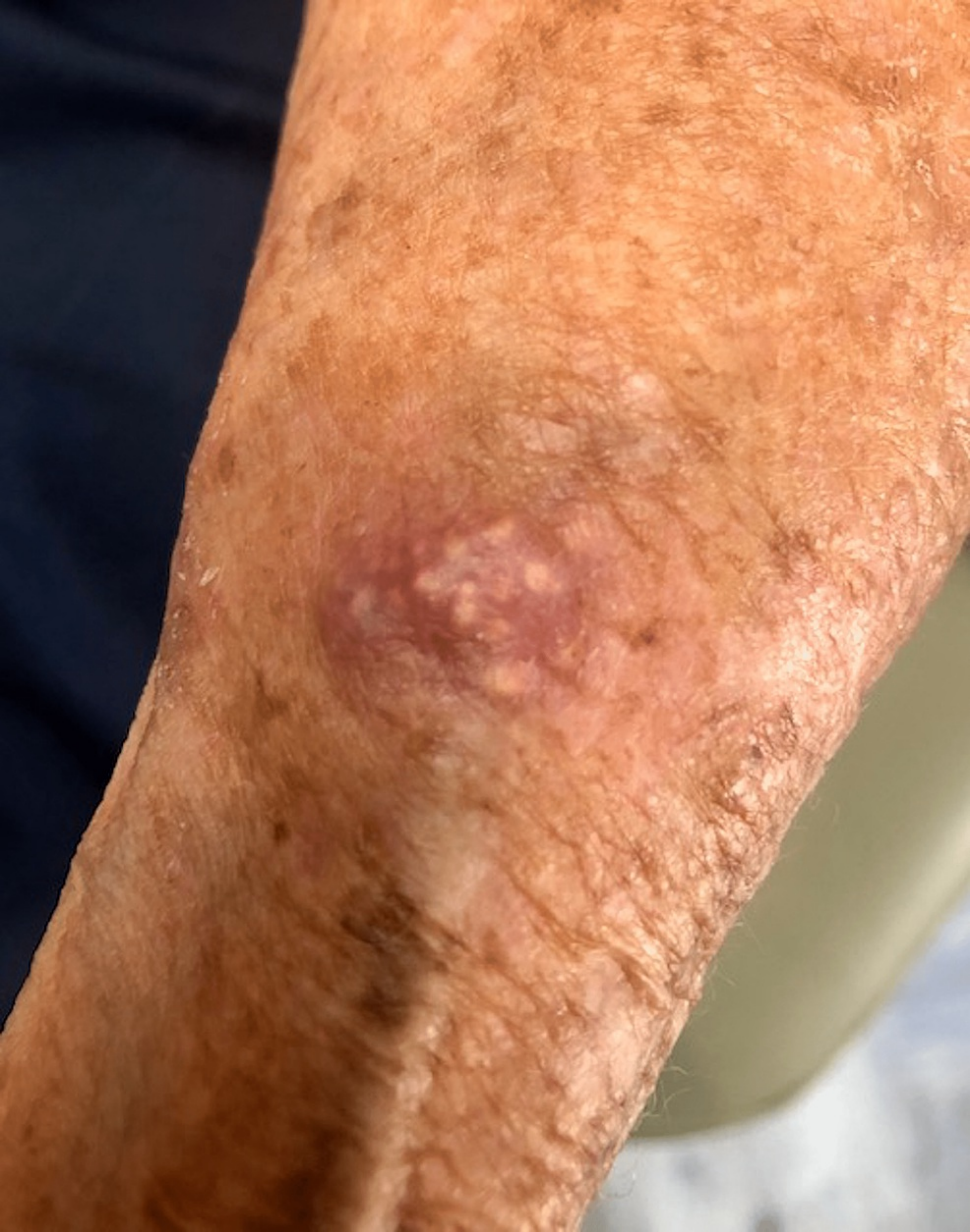
Left dorsal forearm with a 9 mm erythematous papule containing white cystic foci

An 88-year-old female with a history of non-melanoma skin cancer presented to the dermatology clinic with a three-week history of a new, rapidly enlarging but otherwise asymptomatic growth to the left dorsal forearm. She denied any previous inflammation, trauma to the region, or prior skin lesions in that location. She reported no other significant medical history. A physical exam revealed a 9 mm non-tender, erythematous papule with white cystic foci within the lesion (Figure 1).

**Figure 2:**
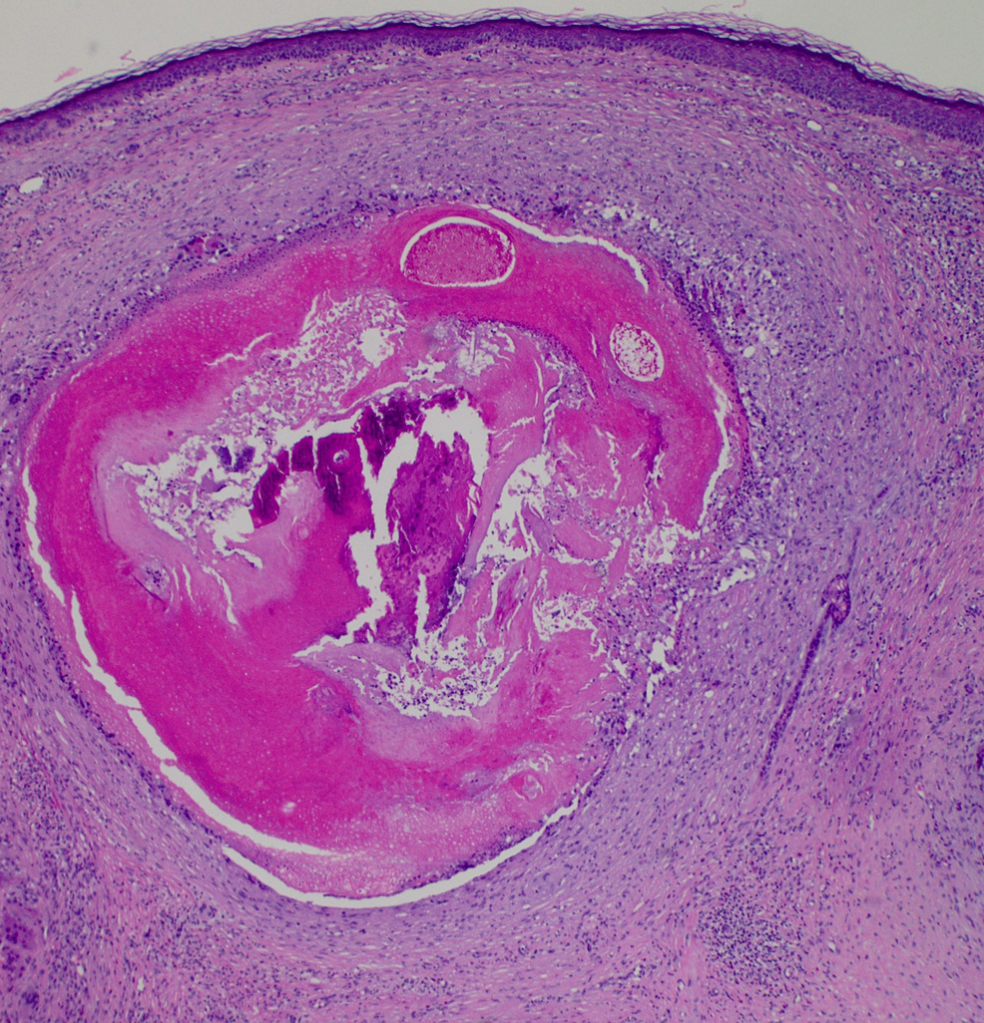
Biopsy stained with hematoxylin and eosin (H&E) at x40 magnification demonstrating a well-circumscribed dermal nodule containing “ghost” or “shadow” cells and a basaloid epithelium

**Figure 3:**
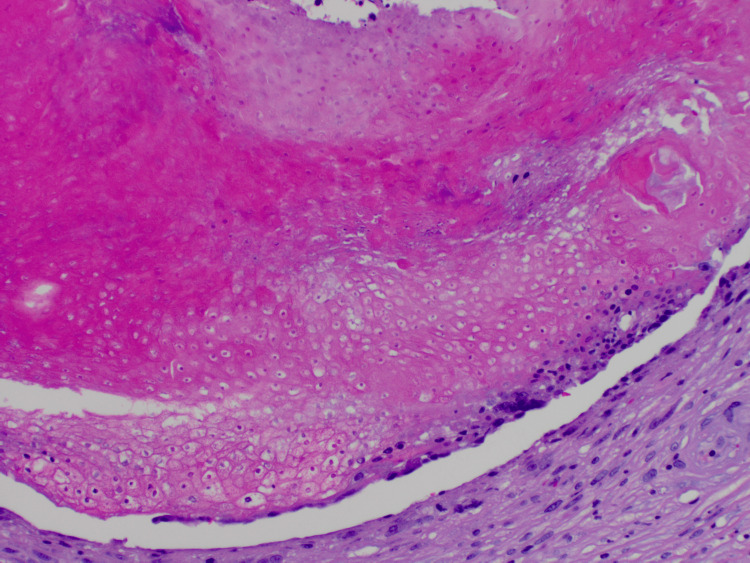
Closer view showing an abrupt transition from basaloid cells to eosinophilic, anuclear “ghost” or “shadow” cells, characteristic of pilomatricomas (H&E, x200 magnification) H&E: hematoxylin and eosin

The clinical differential diagnosis included basal cell carcinoma, squamous cell carcinoma, and calcinosis cutis. Due to its rapid growth and concern for malignancy, a punch biopsy was performed. Pathology showed a well-circumscribed dermal nodule containing “ghost” or “shadow” cells and a basaloid epithelium, with no significant atypia, consistent with a pilomatricoma (Figures 2, 3).

## Discussion

Pilomatricoma is a benign skin tumor of the epithelial hair matrix [1]. It typically presents as a single, slowly enlarging erythematous nodule most commonly located in the head and neck region (especially the face), followed by the upper extremities [2]. Clinically its firmness from secondary calcification may give rise to an angulated shape when the overlying skin is stretched, referred to as the “tent sign” [4]. Histologically, pilomatricomas contain islands of anucleated eosinophilic “shadow” or “ghost” cells surrounded by nucleated small basaloid or germinal (hair matrix) cells [5-8]. When calcification is present, the lesion is usually termed a calcifying epithelioma of Malherbe [5]. The histology of our patient’s tumor showed dystrophic calcification despite its brief history of onset.

Pilomatricomas tend to occur in children and young adults below 30 years of age [6,9,10]. In one review of pilomatricomas, 40% of tumors were diagnosed in children younger than 10 years and 60% within the first two decades of life [6]. There is a slight female predominance [6]. The definitive treatment of pilomatricomas is complete excision with clear margins [11-13]. While exceedingly rare, there have been reported cases of pilomatrical carcinomas arising from previously excised pilomatricomas, necessitating close monitoring of patients with recurrent lesions after excision [7,11].

Pilomatrical carcinoma, or calcified epithelial carcinoma of Malherbe, represents the malignant variant of pilomatricoma. It is a locally aggressive tumor with a higher tendency for local recurrence even after excision with tumor-free margins [13]. Pilomatrical carcinoma frequently presents as a firm, painless, and asymptomatic dermal or subcutaneous nodule, with or without overlying violaceous discoloration and ulceration [14-17]. In contrast to pilomatricomas, pilomatrical carcinomas are more common in males [5]. Pilomatrical carcinomas have a bimodal prevalence of disease within the first three decades and the sixth to seventh decades of life [14-16], the latter representing a distinguishing feature from pilomatricomas. The age of our patient and the rapid growth of her lesion were features more in line with pilomatrical carcinoma, but the histology of her tumor did not suggest malignancy. Histologically, pilomatrical carcinomas are more likely to exhibit a high mitotic rate with atypical mitoses, central necrosis, infiltration of the skin and soft tissue, and invasion of blood and lymphatic vessels [1,18,19].

A comprehensive review of 2189 pilomatricoma cases in the literature found the patients’ mean age of excision to be 16 years and seven months, with a range of five months to 97 years [11]. The oldest patient with pilomatricoma included in that review, reported by Davies et al. in 2016, was a 97-year-old male with a large (3.1 cm), rapidly growing, and recurrent lesion on the eyebrow [5]. The lesion had been present for three months but had more explosive enlargement over a month. It was excised but then recurred about one month after the initial resection [5]. Behnke et al. reported four elderly patients aged 54 to 85 years with histopathologically diagnosed pilomatricoma, but the lesions in all four cases were located on the face (specifically on the eyebrow, cheek, root of the nose, and preauricular area) [3]. None of those reported were located on the upper extremity [3]. Ours represents a unique case of a new-onset pilomatricoma on the dorsal forearm of an 88-year-old female. Based on our literature review, this is the oldest patient with a forearm pilomatricoma reported to date.

Although pilomatricomas are well-recognized lesions, they are frequently misdiagnosed clinically [15]. It is reported that pilomatricomas are accurately diagnosed preoperatively in only 28% to 43% of cases, likely due to their atypical and variable presentation [7].

## Conclusions

We did not initially consider pilomatricoma in our patient based on her older age of onset, the rapid growth of her lesion over a period of three weeks, the unusual clinical findings, and her previous history of skin cancer. However, this case highlights that pilomatricomas are not limited to children and young adults, and when present in elderly patients, may be found in locations outside the face. Dermatologists should consider pilomatricomas in the differential diagnosis of rapidly growing skin lesions on the upper extremities of elderly patients. It is important to confirm this diagnosis with biopsy since the clinical features of pilomatricomas may overlap with those of malignant skin lesions.
